# Characterization of plasmids harboring *bla*_CTX-M_ and *bla*_CMY_ genes in *E*. *coli* from French broilers

**DOI:** 10.1371/journal.pone.0188768

**Published:** 2018-01-23

**Authors:** Fabrice Touzain, Laetitia Le Devendec, Claire de Boisséson, Sandrine Baron, Eric Jouy, Agnès Perrin-Guyomard, Yannick Blanchard, Isabelle Kempf

**Affiliations:** 1 ANSES, Ploufragan Laboratory, Ploufragan, France; 2 Université Bretagne Loire, Rennes, France; 3 ANSES, Fougères Laboratory, Fougères, France; Ross University School of Veterinary Medicine, SAINT KITTS AND NEVIS

## Abstract

Resistance to extended-spectrum cephalosporins (ESC) is a global health issue. The aim of this study was to analyze and compare plasmids coding for resistance to ESC isolated from 16 avian commensal and 17 avian pathogenic *Escherichia coli* (APEC) strains obtained respectively at slaughterhouse or from diseased broilers in 2010–2012. Plasmid DNA was used to transform *E*. *coli* DH5alpha, and the resistances of the transformants were determined. The sequences of the ESC-resistance plasmids prepared from transformants were obtained by Illumina (33 plasmids) or PacBio (1 plasmid). Results showed that 29 of these plasmids contained the *bla*_CTX-M-1_ gene and belonged to the IncI1/ST3 type, with 27 and 20 of them carrying the *sul2* or *tet*(A) genes respectively. Despite their diverse origins, several plasmids showed very high percentages of identity. None of the *bla*_CTX-M-1_-containing plasmid contained APEC virulence genes, although some of them were detected in the parental strains. Three plasmids had the *bla*_CMY-2_ gene, but no other resistance gene. They belonged to IncB/O/K/Z-like or IncFIA/FIB replicon types. The *bla*_CMY-2_ IncFIA/FIB plasmid was obtained from a strain isolated from a diseased broiler and also containing a *bla*_CTX-M-1_ IncI1/ST3 plasmid. Importantly APEC virulence genes (*sitA-D*, *iucA-D*, *iutA*, *hlyF*, *ompT*, *etsA-C*, *iss*, *iroB-E*, *iroN*, *cvaA-C* and *cvi*) were detected on the *bla*_CMY-2_ plasmid. In conclusion, our results show the dominance and high similarity of *bla*_CTX-M-1_ IncI1/ST3 plasmids, and the worrying presence of APEC virulence genes on a *bla*_CMY-2_ plasmid.

## Introduction

Resistance to extended-spectrum cephalosporins (ESC) is encountered in *Enterobacteriaceae* isolated from animal and human origins. In humans, as in companion and farm animals, ESC resistance may be encountered in commensal or pathogenic strains. In France, the prevalence of ESC-resistant (ESCR) *E*. *coli* is monitored in poultry, either by sampling of caeca samples from healthy broilers at slaughterhouse in the framework of the annual national monitoring program, or via the RESAPATH network for pathogenic strains [1]. For commensal *E*. *coli* isolated from broilers, the percentages of ESCR isolates increased from 4% in 2010 to 10.4% in 2012 decreasing to 4.0% in 2014 [2]. Concerning *E*. *coli* isolates obtained from diseased poultry, the highest percentages of ESCR pathogenic *E*. *coli* were observed in 2010 and 2011, with levels above 20% but decreasing to 2.5% in 2015 [1].This decrease was also noticed for other antimicrobials, probably resulting from the Ecoantibio Plan, a public policy set up by the Ministry of Agriculture, Agro-Food and Forestry to reduce the risks of antibiotic resistance used in veterinary medicine. In France, as in several European countries, ESC-resistance is mainly attributed to the production of extended-spectrum beta-lactamases such as CTX-M encoded by conjugative plasmids [3] and to a lesser extent to AmpC-type beta-lactamases [4]. Because plasmids are capable of persistence and diffusion in various bacterial hosts, they are considered as key elements for the spread of ESC resistance. In this context we decided to sequence plasmids from pathogenic and non-pathogenic *E*. *coli* strains isolated from broilers of various production types (organic, conventional or export) and geographic areas in 2010–2012 and containing one of the two most prevalent genes (*bla*_CTX-M_ and *bla*_CMY-2_) [3, 4] to evaluate their diversity and the presence of antimicrobial resistance, virulence or other significant determinants. Thus our aim was to investigate whether plasmids from avian pathogenic *E*. *coli* (APEC) obtained from internal organs and commensal strains obtained from caeca, shared common characteristics and whether similar plasmids could be present in strains isolated from birds from different hatcheries, geographical regions and production types. Moreover the sequencing of the ESC-resistance plasmids from APEC strains gave us the possibility to determine whether virulence and ESC-resistance determinants could be borne on the same mobile genetic element.

## Material and methods

### Characterization of bacterial strains

The bacterial strains were randomly selected from among two collections of *E*. *coli* isolates, one from the national antimicrobial resistance monitoring program based on caeca samples from healthy broilers at slaughterhouse, the other consisting of strains obtained from typical lesions of avian colibacillosis (hereinafter referred to as pathogenic strains), provided by the RESAPATH network. The selection criteria for inclusion in the study were the year of isolation (2010–2012), the host (broiler), the resistance to ESC and the presence of either the group 1 *bla*_CTX-M_ gene, or the *bla*_CMY-2_ gene, as suggested by previous PCR tests [5, 6] in the frame of routine monitoring. Thus 16 commensal and 17 pathogenic strains were included (Table 1). For commensal isolates, the sampling strategy ensured that only one isolate per flock was included. Based on data relative to the type of production, hatchery, French *département* of the farm and slaughterhouse, broiler age (Table 1 and S1 Table) and date of sampling, we included isolates from various non-epidemiologically related flocks. Thus the ESCR commensal isolates (strains COV1 to COV16) originated from conventional (11 strains), export (4 strains) and organic (1 strain) broiler productions, from 8 different hatcheries, and from 11 and 6 French *départements* respectively for farms and slaughterhouses. Birds were 32 to 90 days old. Few data concerning the isolates obtained from colibacillosis lesions of diseased broilers (COV17 to COV33) were available, each strain being from a single flock. No data concerning the pathogenicity of these isolates obtained from diseased birds was available.

**Table 1:** Main characteristics of strains.

Strains	16 commensal strains	17 strains from diseased broilers
Origin of strains	Production type: 11 conventional, 4 export and 1 organic broiler flocks Hatcheries: 8 different hatcheries reported Farms: from 11 French *départements* Slaughterhouses from 7 French *départements* Age of broilers at sampling: 32 to 90 days	Not reported
Resistance to		
AMP	16/16 (100%)^a^*	17/17 (100%)^a^
CTX	16/16 (100%)^a^	17/17 (100%)^a^
FOX	2/16 (12%)^a^	2/17 (12%)^a^
TET	12/16 (75%)^a^	17/17 (100%)^b^
SMX	13/16 (81%) ^a^	17/17 (100%)^a^
CIP	7/16 (44%)^a^	12/17 (70%)^a^
TMP	8/16 (50%)^a^	8/17 (47%)^a^
STR	7/16 (44%)^a^	5/17 (29%)^a^
GEN	1/16 (6%)^a^	0/16 (0%)^a^
KAN	0/16 (0%)^a^	2 (12%)^a^
Phylogenic groups (number of strains)	A (6), B1 (3), C (1), D (1), E (3), F (2)	A (1), B2 (2), C (5), E (2), F (7)

AMP: ampicillin, CTX: cefotaxime, FOX: cefoxitin, TET: tetracycline, SMX: sulfamethoxazole, CIP: ciprofloxacin, TMP: trimethoprim, STR: streptomycin, GEN: gentamicin, KAN: kanamycin

*Percentages in the same row bearing the different superscript letter are significantly different (*P*<0.05)

All strains had been isolated on non-selective media and identified as *E*. *coli* by local veterinary laboratories, and sent to the ANSES laboratories. On arrival at the Ploufragan laboratory, the identity of the strains was checked by PCR [7] and the presence of the *bla*_CTX-M_ or *bla*_CMY-2_ genes was sought by PCR [5, 6].

The isolates were further studied by determination of their phylogenetic groups [8]. Their PFGE or ERIC-PCR patterns were compared [9, 10]. Plasmids were purified according to Takahashi and Nagano [11] and used to transform *E*. *coli* DH5alpha. The transformants were selected on Mueller Hinton media supplemented with cefotaxime (4, 8 and 16 mg/l) for isolates harboring the *bla*_CTX-M_ gene or with cefoxitin (8 and 16 mg/l) for isolates bearing the *bla*_CMY-2_ gene. The susceptibility profiles of the parental strains and of the transformed *E*. *coli* were compared using broth micro-dilution tests with Sensititre plates (Thermo Fisher Diagnostics, Dardilly, France) according to CLSI [12]. The strains were then classified as wild-type (susceptible) or non-wild-type (resistant) according to the epidemiological cut-offs proposed by EUCAST (http://mic.eucast.org/Eucast2/). Plasmids were then purified from the transformants and used for sequencing. A few plasmids were further analyzed by PFGE after S1 nuclease treatment [13].

### Plasmid sequencing

Plasmid DNA was sheared by sonication using a Bioruptor® Plus (Diagenode) apparatus. Libraries were then prepared using NEBNext Ultra DNA library Prep Kit for Illumina and NEBNext Multiplex Oligos for Illumina (index primers Set 1 and Set 2) according to the manufacturer’s instructions (New England Biolabs). Purification steps were conducted with Agencourt AMPure XP magnetic beads (Beckman-Coulter).

Sequencing was performed using Mi-seq Illumina technology (paired-end 2x250 nucleotides) at the Biogenouest (Nantes, France) core facility. Sequences were cleaned with Trimmomatic 0.32 [14] software (ILLUMINACLIP:illumina_oligos_and_reverse_complements:2:30:5:1:true LEADING:3 TRAILING:3 MAXINFO:40:0.2 MINLEN:36 options). Two Bowtie 2 [15] alignments were performed (-non-deterministic -very-sensitive options) on cleaned sequences, one to the *bla*_CTX-M-1_ gene (DQ915955) so as to evaluate sequencing depth and one to phiX174. This second alignment was made to remove reads matching phiX174 material, which is used in Illumina sequencing in case of very redundant samples. The phiX174 unaligned reads were downsampled to fit a global coverage estimation of 80 times. After best kmer size estimation by KmerGenie [16], SPAdes 3.1.1 (20) and MIRA 4.0rc1[17] assemblers were run on Trimmomatic [14] cleaned reads and raw reads, respectively. Redundant or poorly covered contigs were filtered out of assemblies. We retained only the best assemblies based on N50s and lengths.

As a total of 92 contigs was obtained after Illumina sequencing for the pCOV24 plasmid, we decided to use the PacBio sequencer to improve the sequence data obtained for this plasmid. Library preparation and sequencing was performed according to the manufacturer’s instructions “Shared protocol-20kb Template Preparation Using BluePippin Size Selection system (15kb size Cutoff)”. At each step DNA was quantified using the Qubit dsDNA HS Assay Kit (Life Technologies). DNA purity was tested using the nanodrop (Thermofisher) and size distribution and degradation assessed using the Fragment analyzer (AATI) High Sensitivity NGS Fragment Analysis Kit. Purification steps were performed using 0.45X AMPure PB beads (Pacbio).

10μg of DNA were purified then sheared at 40kb using the meraruptor system (diagenode). A DNA and END damage repair step was performed on 5μg of sample. Then blunt hairpin Adpters were ligated to the library. It was treated with an exonuclease cocktail to digest unligated DNA fragments. A size selection step using a 11kb cutoff was performed in the BluePippin Size Selection system from Sage Science with the 0.75% agarose cassettes, Marker S1 high Pass 15–20kb.

Conditionned Sequencing Primer V2 was annealed to the size-selected SMRTbell. The annealed library was then bound to the P6-C4 polymerase using a ratio of polymerase to SMRTbell at 10:1. Then after a magnetic bead-loading step, SMRTbell libraries were sequenced on RSII instrument at 0.2nM with a 360 min movie.

One SMRTcell was used for sequencing this library. Sequencing results were validated and provided by the Integrated next generation sequencing storage and processing environment NG6 accessible in our genomic core facility website [18].

Assembly was performed following Pacific Biosciences recommendations, using HGAP assembler, on the longest reads (minimum seed read length set to 6000, genome size to 5,000,000, overlapper min length to 40, and target coverage to 15). This provided us with the complete *E*. *coli* DH5alpha bacterial chromosome and its expected plasmid.

The resulting plasmid assemblies were submitted to ResFinder to identify resistance genes, to PlasmidFinder and to the pMLST webtool (https://cge.cbs.dtu.dk/services to characterize plasmid sequences, and to the RASTA-Bacteria program version 2.12 (http://genoweb1.irisa.fr/duals/RASTA-Bacteria/) to identify putative toxin-antitoxin systems.

Sequences of virulence genes (*sitA-D* (an ABC iron transport system, >gi|84060731:91375–92289, >gi|84060731:92289–93116, >gi|84060731:93113–93949, >gi|84060731:93968–94825), *iucA-D* (aerobacterin, another siderophore system, >gi|84060731:98089–99870, >gi|84060731:99871–100818, >gi|84060731:100818–102560, >gi|84060731:102557–103834), *iutA* (ferric aerobactin receptor, >gi|84060731:103916–106117), *hlyF* (avian hemolysin, hlyF >gi|84060731:c113559-111806), *ompT* (outer membrane protease, >gi|84060731:c115274-114321), *etsA-C* (*E*. *coli* ABC transport system, >gi|84060731:119488–119866, >gi|84060731:119862–121802, >gi|84060731:121806–123176), *iss* (increased serum survival and complement resistance, >gi|84060731:137931–138239), *iroB-E* (siderophore salmochellin, >gi|84060731:141336–142499, >gi|84060731:142513–146298, >gi|84060731:146402–147631, >gi|84060731:147716–148672), *iroN* (siderophore receptor, >gi|84060731:c150894-148717), *cvaA-C* (Colicin V secretion proteins, >gi|84060731:155164–156075, >gi|84060731:156050–158164, >gi|84060731:c158645-158334), *cvi* (Colicin V immunity protein, >gi|84060731:c158859-158623), *tsh* (temperature-sensitive hemagglutinin, >gi|84060731:c170806-166673), *eitA-D* (*E*. *coli* ABC iron transporter, >gi|84060731:176416–177414, >gi|84060731:177414–178451, >gi|84060731:178448–179212, >gi|84060731:179134–180456)) which may be present on plasmids of avian pathogenic *E*. *coli* [19] were sought.

For each plasmid group, global alignment outputs of progressiveMauve (25) were converted in multi-fasta with the Harvest suite (26). An inhouse script computed percentages of identity for all sequence pairs and for all sequences simultaneously.

### Statistical tests

Distributions were compared using the chi-square test or Fisher’s exact test (http://marne.u707.jussieu.fr/biostatgv/?module=tests/fisher) when the numbers of isolates were inferior or equal to 5. For all tests, values of *P*<0.05 were considered statistically significant differences.

### Accession numbers

BioProject number (PRJNA387700): the BioSample numbers are provided bellow.

Pacbio DH5alpha *Escherichia coli* strain: SAMN07197446.

Pacbio pCOV24 plasmid: pCOV24 (SAMN07197432).

Mi-seq plasmids: pCOV1 to pCOV33: SAMN07197409 to SAMN07197431 and SAMN0719740933 to SAMN0719740942 (see S2 Table for details)

## Results and discussion

### Phenotypic characterization of isolates

All the included strains had reduced susceptibility to ampicillin and cefotaxime. Isolates COV1, COV9, COV28 and COV32 were also non-susceptible to cefoxitin. Resistance to tetracycline and to sulfamethoxazole was very frequently observed (Table 1) (respectively 12/16 (75%) for commensal and 17/17 (100%) for pathogenic strains for tetracycline (*P* = 0.04) and respectively 13/16 (81%) for commensal and 17/17 (100%) for pathogenic strains for sulfamethoxazole (*P* = 0.10)). The other resistances are presented in Table 1. No significant association between the resistances of the commensal strains and the antimicrobials used in the flocks could be observed (data not shown).

The strains belonged to phylogenetic groups A, B1, B2, C, D, E, and F (Table 1). The only two strains belonging to the phylogroup B2 had been isolated from colibacillosis lesions, confirming that this group is associated with extra-intestinal virulence [20]. According to phylogenetic groups, PFGE and ERIC-PCR patterns, the 33 strains were different, except the strains COV26 and COV29 which could not be distinguished by these methods. The diversity of extended spectrum beta-lactamases (ESBL) or pAmpC-producing *E*. *coli* has already been shown by ourselves [3] and others: thus Day et al [21] analyzed 353 ESBL-producing *E*. *coli* isolated from infections in humans or in animals, commensal isolates from animals, and isolates from food of animal origin from the UK, Netherlands and Germany. The authors identified 158 different sequence types (ST) for the *E*. *coli* hosts, with some of them (ST131, ST10, ST88) detected in different animal species.

### Beta-lactamase genes

PCR results confirmed the presence of the *bla*_CTX-M-group1_ gene in 14/16 commensal and 17/17 pathogenic strains whereas the *bla*_CMY-2_ gene was detected in two commensal strains (COV1 and COV9) and in two pathogenic strains (COV28 and COV32); COV28 and COV32 had both genes (Table 2 and S3 Table). The presence of the *bla*_CMY-2_ gene is probably responsible for the cefoxitin resistance observed in COV1, COV9, COV28 and COV32 isolates. The dominance of the *bla*_CTX-M-1_ gene has already been reported in commensal isolates from French broilers [3] and layers [4], but there is limited data available concerning pathogenic isolates to date. Similarly, in the study of Day et al [21] on 353 ESBL-producing *E*. *coli* from three European countries, *bla*_CTX-M-1_ was overall the most frequently detected ESBL gene, followed by *bla*_CTX-M-15_, which was the most common ESBL gene in the human isolates. In their study, the *bla*_CTX-M-1_ gene was also by far the most frequent ESBL gene in isolates of poultry origin. Inversely, in some countries such as Norway, Sweden and Denmark [22], broilers and their products are mainly contaminated by CMY-2 beta-lactamase producing *E*. *coli*. In the Netherlands, various ESBL and AmpC genes have been described in poultry, their environment and their products, the major ones being *bla*_CMY-2_, *bla*_CTX-M-1_, *bla*_SHV-12_ and *bla*_TEM-52_. Other, different beta-lactamase genes such as *bla*_CTX-M-32_ in Italy, *bla*_CTX-M_ group 9 in Spain, *bla*_CTX-M-15_ in the UK, *bla*_CTX-M-55_, *bla*_CTX-M-14_ and *bla*_CTX-M-65_ in China and *bla*_CTX-M-25_ in Japan are also encountered in ESCR *E*. *coli* from poultry in other countries [23]. In other animal species such as pigs and cattle, the same ESBL are usually reported, with notably a very high prevalence of *bla*_CMY-2_ in the Americas and Asia [23].

**Table 2:** Main characteristics of sequenced plasmids.

	ESCR gene	Other ARG	Contigs	Cumulative contig size (bp)	Replicon and pMLST	Addiction systems
Plasmids from commensal strains	*bla*_CTX-M-1_: 13^a^	*tet*(A): 8 *sul2*: 13 *bla*_TEM1b_: 1 *dfr1a*:1 *dfra17*: 3 *aadA5*:3	1–31 contigs (mean: 6.2)	Mean: 119 kbp	IncI1 99.30%: G57A: 13 ST3 100%: 13	ParE-RelB: 11
	*bla*_CMY-2_: 2	-	2 or 6 contigs	Mean: 100 kbp	B/O/K/Z 98% or 97%	RelE-StbD: 1
Plasmids from diseased broilers	*bla*_CTX-M-1_: 17	*tet*(A): 14 *sul2*: 16 *bla*_TEM1b_: 1 *dfra17*: 2 *aadA5*:2	1–27 (mean: 6)	Mean: 110 kbp	IncI1 99.30%: G57A: 13 ST3 100%: 12^b^	ParE-RelB: 12 CcdB-CcdA / COG5302: 1
	*bla*_CMY-2_: 1	-	12	203 kpb	FIA, 100% 384/388 FIB 99.56%, FIC/FII 95.59% F18:A6:B42	VagC/ SpoVT_AbrB/ MazE-VapC/ PIN

^a^number of plasmid

^b^the entire *ardA* gene could not be found in pCOV25, but the four other alleles (*pilL_2*, *repI1_2*, *sogS_1* and *trbA_4*) were those of the pMLST3

### Preparation and characterization of transformants

Transformation experiments led to transformants harboring the *bla*_CTX-M_ or the *bla*_CMY-2_ gene of the parental isolate for all but one isolate (COV8). Although the transformation was repeated a number of times, no transformant could be obtained for COV8 harboring the *bla*_CTX-M-1_ gene, suggesting that this gene was probably borne on the chromosome, but no attempt to confirm this localization was attempted. Chromosomal location of *bla*_CTX-M_ genes has previously been evidenced in *E*. *coli* but the plasmid location of the *bla*_CTX-M_ gene is largely predominant [24].

For the pathogenic strain COV28, two transformants were obtained, one on cefotaxime-supplemented media and bearing the *bla*_CTX-M_ gene and one on cefoxitin-supplemented media and harboring the *bla*_CMY-2_ gene. We were then able to show (see below) that the *bla*_CTX-M_ and the *bla*_CMY-2_ genes were carried by an IncI1 and an IncF plasmid respectively. Similarly, de Been et al [25] described an *E*. *coli* strain from chicken meat containing both an IncI1 and an IncK plasmid carrying the *bla*_CTX-M-1_ and the *bla*_CMY-2_ genes respectively. For the COV32 strain, transformation was repeated a number of times on cefotaxime-supplemented and cefoxitin-supplemented media, but we could only obtain transformants containing the *bla*_CTX-M-1_ gene, suggesting that the *bla*_CMY-2_ gene was located on the chromosome; we did not try to confirm this localization.

Susceptibility tests of the transformants showed that they shared the ESC-resistance of their parental strain and that only the three transformants, obtained from COV1, COV9 and COV28 bearing the *bla*_CMY-2_ gene, were resistant to cefoxitin. In addition to the beta-lactam resistance, other resistances could be transferred, such as resistance to sulphamethoxazole observed in 23 transformants, to tetracycline (23 transformants) and to trimethoprim (6 transformants). No fluoroquinolone, streptomycin, kanamycin or gentamicin resistances were detected in any of the transformants. Interestingly, no resistance other than ESC-resistance was transferred with the plasmids carrying the *bla*_CMY-2_ gene. Such paucity or absence of resistance genes on *bla*_CMY-2_-carrying-plasmids was also observed by others, either on *Salmonella* [26] or on *E*. *coli*: thus among 93 CMY-2-encoding plasmids of *E*. *coli* isolates from humans, poultry meat, poultry and dogs from Denmark, only 4% co-transferred resistance to antibiotics other than beta-lactams [27]. This phenomenon suggests that the selection and persistence of such *bla*_CMY-2_-containing plasmids is probably largely dependent on the use of beta-lactams, whereas *bla*_CTX-M_ plasmids containing resistance to other antibiotic families (tetracyclines, sulphonamides, trimethoprim, etc.) may also be co-selected by non-beta-lactam antibiotics [28]. This could be a reason why the *bla*_CMY-2-_carrying plasmids are less prevalent than *bla*_CTX-M_ ones.

### Sequencing of the plasmids

We retained two SPAdes assemblies and 28 MIRA “LargeContigs” with an average length and N50 statistics of 115309 and 80825 nucleotides, respectively. Lengths ranged from 91138 (COV1) to 115593 nucleotides (COV11) and N50s ranged from 12141 (COV20) to 115593 nucleotides (COV11).

HGAP assembly was retained for the COV24 sample.

### Analysis of plasmid sequences

Transformation of a recipient strain is usually the preferred method before plasmid sequencing, because it lowers the possibility that two or more plasmids enter the recipient cell together. However, plasmid transformation may introduce structural modifications in the plasmid sequence compared to the native one. This must be kept in mind when analyzing and comparing the plasmid sequences. The main features of the plasmid sequences are presented in Table 2. Thirty *bla*_CTX-M_ plasmids were sequenced either by Illumina (29 plasmids) or by Illumina and PacBio (COV24). A unique contig could be obtained for 14 plasmids and their size ranged from 105,870 bp (pCOV31) to 115,993 bp (pCOV11), except for pCOV24 sequenced by PacBio, which contained 131,672 bp. Two to five contigs were obtained for 4 other plasmids, but as many as 31 were obtained for pCOV6 (171,865 bp). The large sizes of pCOV6 and pCOV24 were confirmed by PFGE after S1 nuclease treatment.

#### Sequences of the *bla*_CTX-M- 1_ plasmids

All the 30 *bla*_CTX-M_ plasmids were of the IncI1 replicon type and all shared the same G57A mutation compared to the sequence found in the *Salmonella* enterica subsp. enterica serovar Typhimurium plasmid R64 DNA sequence (accession number AP005147). This G57A mutation was also present in the sequence of the previously published pC49-108 plasmid of a chicken *E*. *coli* isolate (accession number KJ484638) [29]. All 30 IncI1 plasmids were of the pMLST3 type except pCOV25, for which the entire *ardA* gene could not be found, but the four other alleles (*pilL_2*, *repI1_2*, *sogS_1* and *trbA_4*) were those of the pMLST3. In the study by Day et al [21] relative to 353 ESBL-producing *E*. *coli* from humans, animals and food in Germany, the Netherlands and the UK, the most common plasmid replicon type among 341 transformants was also IncI1 followed by multiple IncF replicons, and they observed that IncI1 was also more common in poultry in the Netherlands. They could assign 128 IncI1 plasmids to sixteen different pMLST, with pMLST7 (34%) and pMLST3 (21%) being the most common. Smith et al [30] determined the pMLST of 251 IncI1 plasmids isolated from 2005 to 2009 from humans, poultry and other animal species, and sequenced 32 of them. They showed that IncI1 plasmids containing *bla*_CTX-M-1_ could be assigned to 14 different ST but most belonged to ST7 (70%) or to ST3 (27%). In France however, a high prevalence of *bla*_CTX-M-1_ IncI1/ST3 plasmids was found in animals (cat, dog, goat, pony, horse) [31], in the fecal flora of healthy humans (85%) and in diseased patients (60%) and similarly distributed among adults and children [32].

The genes coding for resistance to beta-lactams (*bla*_CTX-M-1_, all 30 plasmids, *bla*_TEM-1B_, one plasmid (pCOV4)), tetracyclines (*tet*(A), 20 plasmids), sulphonamides (*sul2*, 27 plasmids), trimethoprim (*dfrA1*, one plasmid, *dfrA17*, five plasmids) and aminoglycosides (*aadA5*, five plasmids) were detected and were in agreement with the resistance profiles of the transformants (except for pCOV19, for which no tetracycline-resistance gene could be detected). The *aadA5*, *dfrA1* and *dfrA17* genes have already been detected on *bla*_CTX-M_ IncI1/ST3 plasmids from humans and chickens in Switzerland [33] and the Netherlands [30], *sul2* on *bla*_CTX-M-1_ IncI1 plasmids from *E*. *coli*, *Klebsiella* and *Salmonella* from pigs in the UK in *Salmonella* typhimurium [34]. It is worthy of note that the resistance genes that are most often carried on the *bla*_CTX-M_ plasmid are resistances to tetracyclines and trimethoprime-sulphonamides, antimicrobials that are frequently used in poultry production [35].

All IncI1 plasmids had the conjugative transfer genes *pilI-T*, *pilV*, *traA-C*, *traE-F*, *traH*, *traJ-X*, the DNA primase and endonuclease genes. They also shared the genes involved in maintenance and stability regions. As an example, the circular representation of plasmid pCOV4 and comparison with two other *bla*_CTX-M-1_ IncI1/ST3 plasmids, pCOV5 and pCOV18, is given in S1 Fig.

None of the APEC plasmidic virulence genes could be detected on the *bla*_CTX-M-1_ plasmids, although, according to hybridization on a micro-array [36] performed for some of these *bla*_CTX-M-1_-containing strains, the *iroN* gene had been detected on the whole genome of four out of eight tested commensal strains and on all 14 tested pathogenic ones. Similarly, the *iss* gene had been detected on seven out of eight commensal strains and on 14 out of 14 pathogenic ones. It is thus probable that, in addition to the *bla*_CTX-M-1_ IncI1/ST3 ESCR plasmid, these strains contain another plasmid encoding APEC virulence genes.

According to RASTA, no putative toxin-antitoxin systems could be detected in seven plasmids, 21 had the ParE-RelB system and one (pCOV18) had the CcdB-CcdA/COG5302 system.

The full 1263 bp IS*Ecp1* (accession number AJ242809) could be detected in 27 plasmids, and a partial one was detected in pCOV3 and pCOV20. The distance between IS*Ecp1* and the *bla*_CTX-M-1_ gene could be estimated for 24 plasmids, when the two sequences were obtained on the same contig: 288 bp for 20 plasmids, 1,488 bp for five of them (pCOV10, pCOV11, pCOV12, pCOV15 and pCOV24) and 27,531 bp for pCOV4.

A putative transposase of 1181 bp was present in 22 plasmids and was identical to the transposase previously reported in other plasmids such as pC49-108 (accession number KJ484638, region: 102662–103842) [29] or p369 (accession number KT779550, region: 87786–88966) [37]. This transposase was found to be located at 19–22 kbp from the *bla*_CTX-M-1_ gene. The 806 bp IS26 was present on 26 plasmids at approximately 24.6 to 30 kpb from the *bla*_CTX-M-1_ gene. A 1352 nt integrase identical to IntI1 (accession number KC340960, region: 9704–10714) was found on five plasmids (pCOV4, pCOV10, pCOV11, pCOV12 and pCOV24). The distance between the integrase and the *bla*_CTX-M-1_ gene was greater than 22 kbp and 27 kbp on pCOV10 and pCOV24 respectively. A 616 nt portion of Int1 was detected on pCOV20.

Comparison of the sequences of the 30 *bla*_CTX-M_-containing plasmids according to progressiveMauve for the 114,313 common nt (but alignment could contain gaps), showed that overall they shared 66.83% identity. Interestingly, two plasmids, pCOV2 and pCOV7, showed 100% identity although their origin (production types, hatcheries, regions and slaughterhouses) differed as well as the characteristics of the parental strain such as phylogenetic groups and antimicrobial resistances (Table 1). Other plasmids such as pCOV16 from a commensal strain and pCOV17, pCOV18, pCOV22, pCOV27, pCOV30, pCOV31 and pCOV33 shared more than 99% identity although the susceptibility and/or phylogenetic groups and, when available, virulence profile [36] of their parental strains were different. A high level of similarity between IncI1/ST3 plasmids from human or chicken meat origins was previously reported by de Been et al [25], who observed that a maximum of only four SNP was found in the 40 kbp plasmid core of their subset of IncI1/ST3 plasmids.

Thus globally, the results obtained showed that, considering the *E*. *coli* commensal or pathogenic isolates obtained from French broilers, the *bla*_CTX-M-1_ gene was most often harbored by IncI1 ST3 plasmids, and these highly similar plasmids were found in broilers of different production types (conventional, export or organic), issued from different hatcheries and bred in ten different French *départements*. This low diversity is probably in relation with the vertical transmission of ESCR *E*. *coli* strains from breeders to broilers [38] associated with diffusion of the resistance plasmids between *E*. *coli* strains and probably a high fitness and adaptability of such plasmids, which are encountered in *E*. *coli* hosts from different environments (intra- or extra-intestinal) and various animal hosts.

#### Sequences of the *bla*_CMY-2_ plasmids

The three *bla*_CMY-2_ plasmids were obtained from two commensal (COV1, COV9) and one pathogenic (COV28) strains, the latter having two different plasmids, one containing *bla*_CTX-M-1_ (pCOV28A) and another with *bla*_CMY-2_ (pCOV28B). Contrary to the *bla*_CTX-M_ plasmids, the *bla*_CMY-2_ gene was borne on IncB/O/K/Z-like replicons (pCOV1 and pCOV9) or on an IncFIA/FIB plasmid (pCOV28B).

For the IncB/O/K/Z-like replicons, differences (G6A, T10C, C135G for pCOV1 and pCOV9 and G13A for pCOV9) were observed in the RNAI target sequence used for the replicon typing, compared with the reference plasmids recently reported by Seiffert et al [39] for the IncK2 group. Similarly, for the pCOV28B plasmid, mutations were detected in the target genes (first four nucleotides of the FIA target and mutations A30G, T244A and T607C in the FIB target).

In the study of Hansen et al [27] on plasmids encoding CMY-2 beta-lactamase in *E*. *coli* from humans, poultry meat, poultry and dogs in Denmark, the *bla*_CMY-2_ gene was mainly detected on IncI1 and IncK plasmids, whereas the IncA/C and IncFII were rarely evidenced (3% and 1% respectively). IncI1 was more frequent in human and animal isolates and IncK dominated in isolates from Danish and imported broiler meat. Other studies reported that *bla*_CMY-2_ were mainly found on IncI1 and IncA/C plasmids, with IncA/C predominant in the USA, and the gene was rarely found on IncK, IncF and colE plasmids [40]. It is thus noteworthy that none of our *bla*_CMY-2_ plasmids belonged to IncI1 or IncA/C replicon types.

In accordance with the absence of transfer to the recipient cell of resistance to other antimicrobial families, none of the three *bla*_CMY-2_ plasmids contained other antimicrobial resistance genes. Similarly, in the study of Hansen et al [27], co-transfer of other resistances was very rarely observed. In this latter study, twelve *bla*_CMY-2_ IncK plasmids were sequenced; their size ranged from 79,431 to 90,439 bp, and a maximum of 14 SNP was observed in the 76,863 bp identified in all but one of the sequenced plasmids. A similar finding was reported by de Been et al, [25] whereas we found only 78.93% identity in the 95,707 nt from pCOV1 and pCOV9 that could be aligned.

Conjugative transfer regions (*tra*, *trb* and pili operons), genes involved in the inhibition of SOS response (*psiAB*), were also detected on pCOV1 and pCOV9; the *bla*_CMY-2_ was flanked by an ISEcp1 and *blc-sugE* elements, as in pCOV1 and pCOV9, and as described by others [22, 41, 42]. According to RASTA, pCOV9 contained the RelE-StbD system, but no putative toxin-antitoxin system was detected in pCOV1. No APEC virulence gene was detected on pCOV1 and pCOV9 plasmid sequences, although the *iroN* and the *iss* genes had been detected in the parental strains [36].

pCOV28B belonged to the F18:A6:B42 sequence type. It contained the ISEcp1. The IncF plasmid conjugative transfer genes (*tra*A-*traN*, *traP-traR*, *traT-traY* and *trbA-trbJ*) were present, as well as the toxin-antitoxin VagC/SpoVT_AbrB/MazE-VapC/PIN systems. Importantly, this plasmid, isolated from a bird suffering from colibacillosis, contained the APEC virulence genes *sitA-D*, *iucA-D* and *iutA* on contig 7, *hlyF* and *ompT* on contig 5, *etsA-C*, *iss* and *iroB-E* on contig10, *iroN* (2133pb on contig 1 and the remaining part on contig10), *cvaA-C* and *cvi* (encoding colicinV) on contig 9, but not the *tsh* or the *eitA-D* genes. Thus the five genes (*iutA*, *hlyF*, *ompT*, *iss* and *iroN*) that can be used as minimal predictors of APEC virulence [43] were detected. Johnson et al showed that several putative virulence traits, including *iss*, *tsh*, iron acquisition and transport systems including those encoding aerobactin and salmochelin, the *sit*ABC iron transport system, and *eit*, a putative iron transport system novel to APEC, were located on a cluster on the pAPEC-O2-ColV (accession no. AY545598.5) plasmid [44]. Later, they also reported that the virulence could also be borne by a cluster on pAPEC-O1-ColBM (accession number DQ381420), which contains a putative virulence cluster similar to that of pAPEC-O2-ColV and encodes colicins B and M, not encoded by pAPEC-O2-ColV [19]. The genes encoding these colicins B and M (*cma*, *cmi*, *cba* and *cbi*) were also present on contig 3 on pCOV28B. Wang et al studied a multi-drug-resistant (but not ESCR) APEC strain isolated in China and concluded that the virulence genes and the resistance genes were on different plasmids [45]. Although sequence data showed that the *bla*_CMY-2_ gene and the different virulence genes were detected on different contigs, respectively contig 2 for the *bla*_CMY-2_ gene and contigs 1, 3, 5, 7, 9 and 10 for the virulence ones, the mean coverages for these different contigs ranging from 459 to 490, strongly suggest that the ESC-resistance gene is on the virulence plasmid. Furthermore PFGE after S1 nuclease treatment showed the presence of a unique plasmid of the expected size (203 kbp). The presence of both ESC resistance genes and APEC virulence genes on the same mobile genetic element is worrying for public health, as some APEC may cause human infections [46].

## Conclusion

Thirty-three plasmids containing either *bla*_CTX-M_ or *bla*_CMY_ genes, obtained from ESCR *E*. *coli* strains isolated in 2010–2012 from French broilers at slaughterhouse or from lesions of avian colibacillosis, were sequenced. The results showed that 29/30 *bla*_CTX-M-1_ containing plasmids belonged to the IncI1, ST3 type and many harbored the sulphonamides *sul2* (27 plasmids) and/or the tetracycline *tet*(A) (20 plasmids) resistance genes, whereas other genes coding for trimethoprim or aminoglycosides were less often detected. None of these IncI1, ST3 *bla*_CTX-M-1_ plasmids contained APEC virulence genes, although some of them were detected in the parental strains. In spite of their diverse origins, a high level of similarity between these IncI1, ST3 plasmids was evidenced. Inversely three *bla*_CMY-2_ plasmids contained no other resistance genes and were IncB/O/K/Z-like or IncFIA/FIB replicons. The presence of APEC virulence genes on the *bla*_CMY-2_ IncF plasmid obtained from a diseased bird is worrying for animal and public health.

## Supporting information

S1 Fig Main features of plasmids pCOV4, pCOV5, and pCOV18. (TIF)

S1 Table Characteristics of strains and their transformants. (DOCX)

S2 Table Accession numbers. BioProject number (PRJNA387700): the BioSample numbers are provided bellow.
(DOCX)

S3 Table Characteristics of sequenced plasmids. (DOCX)
