# Supplementary figures and images for: Characterization of plasmids harboring blaCTX-M and blaCMY genes in E. coli from French broilers
Source: PLoS One. 2018 Jan 23;13(1):e0188768. doi: 10.1371/journal.pone.0188768 (PMC5779644; doi:10.1371/journal.pone.0188768)

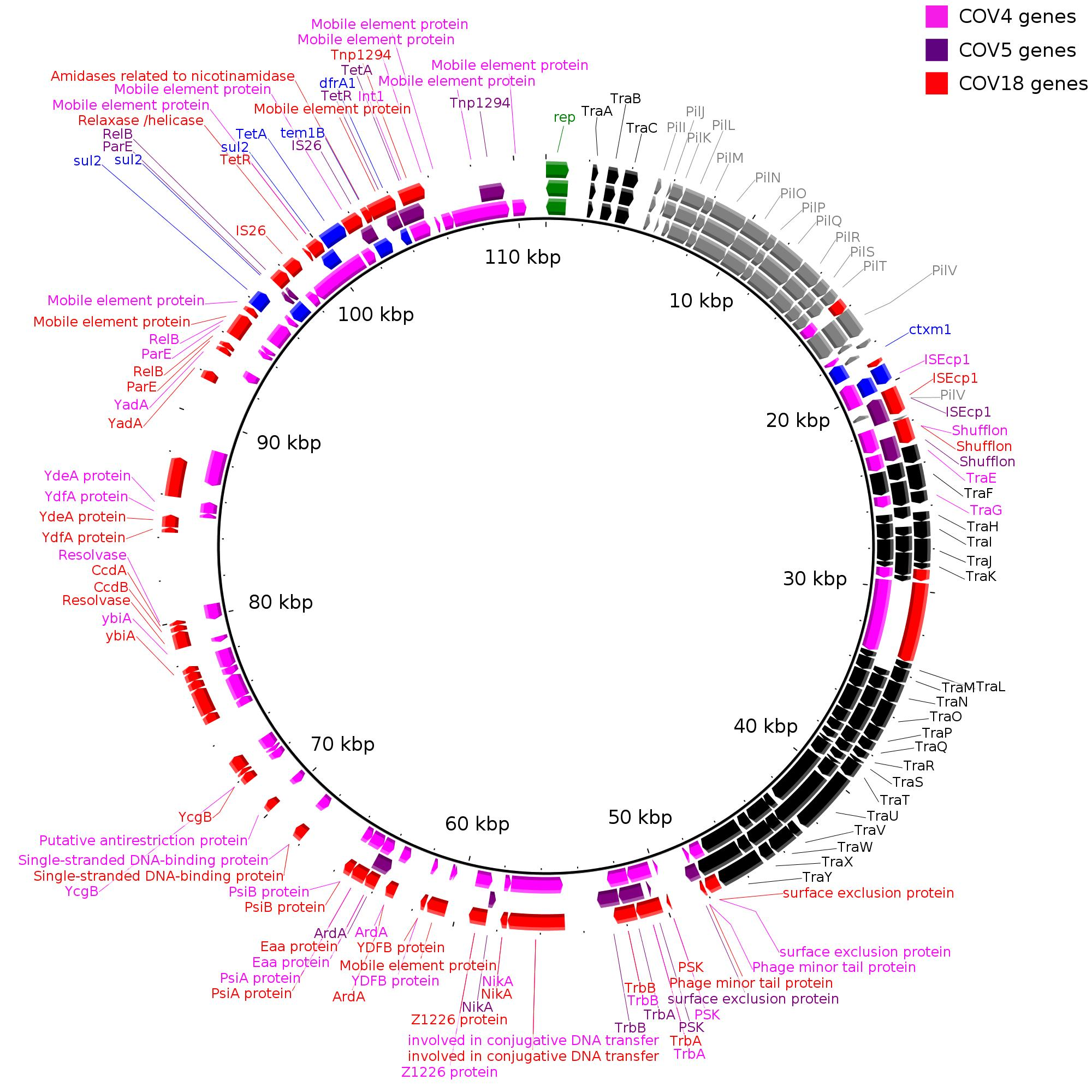

Supplement: S1 Fig — (TIF) [file pone.0188768.s001.tif]
