# Supplementary material for: Characterization of plasmids harboring blaCTX-M and blaCMY genes in E. coli from French broilers
Source: PLoS One. 2018 Jan 23;13(1):e0188768. doi: 10.1371/journal.pone.0188768 (PMC5779644; doi:10.1371/journal.pone.0188768)
Supplement: S1 Table — (DOCX) [file pone.0188768.s002.docx]

**S1 Table. Characteristics of strains and their transformants**

| Strain | Gallus Type^a^ | Hatchery code | Farm^b^ | Slaugherhouse^b^ | Age^c^ | Treatments^d^ | Healthy/pathology | Phylogenetic Group | Other resistances^f^ | ARG^h^ in transformants | | | | |
| --- | --- | --- | --- | --- | --- | --- | --- | --- | --- | --- | --- | --- | --- | --- |
|  |  |  |  |  |  |  |  |  |  | *bla* _CTX-M-1_ | *bla* _CMY-2_ | *tet*(A) | *sul2* | Other ARG |
| COV1 | C | NR | NR | G | NR |  | H | F | FQ- | - | + | - | - | - |
| COV2 | O | 6 | C | C | 90 |  | H | E | TET-SMX- | + | - | + | + | - |
| COV3 | C | 3 | E | L | NR | SXT | H | D | FQ-TET-TMP-SMX-STR | + | - | + | + |  |
| COV4 | C | 8 | I | F | 33 | SXT | H | E | FQ-TET-TMP-SMX-STR | + | - | - | + | *bla*_TEM1b_, *dfra1* |
| COV5 | E | 2 | J | K | 32 | FQ AMP COL | H | E | FQ-TET-SMX-STR-GEN | + | - | + | + | - |
| COV6 | C | 5 | H | B | 37 |  | H | A | TET-SMX | + | - | + | + | - |
| COV7 | C | 1 | A | B | NR |  | H | C | FQ-TET-TMP-SMX-STR | + | - | + | + | - |
| COV8^g^ | E | 2 | D | K | 32 | FQ AMP | H | B1 | FQ^g^ | NA | NA | NA | NA | NA |
| COV9 | C | 7 | G | I | 40 |  | H | A | TET- | - | + | - | - | - |
| COV10 | C | 7 | G | I | 37 |  | H | B1 | TMP-SMX- | + | - | - | + | *dfra17, aada5* |
| COV11 | E | 2 | K | K | 34 |  | H | B1 | TMP-SMX-STR | + | - | - | + | *dfra17, aada5* |
| COV12 | E | 2 | K | K | 33 |  | H | A | TET-TMP-SMX | + | - | - | + | *dfra17, aada5* |
| COV13 | C | 5 | H | I | 38 |  | H | A | FQ-TET-TMP-SMX-STR | + | - | + | + | - |
| COV14 | C | 4 | L | L | 53 |  | H | A | TET-TMP-SMX-STR | + | - | + | + | - |
| COV15 | C | 5 | B | B | 37 | AMX | H | A | TET-SMX | + | - | - | + | - |
| COV16 | C | 3 | A | B | 39 | DOXY | H | F | TET-SMX | + | - | + | + | - |
| COV17 | NR | NR | NR | NR | NR | NR | P | C | FQ-TET-TMP-SMX-STR | + | - | + | + | - |
| COV18 | NR | NR | NR | NR | NR | NR | P | A | TET-SMX | + | - | + | + | - |
| COV19 | NR | NR | NR | NR | NR | NR | P | C | FQ-TET-TMP-SMX | + | - | - | (+) | - |
| COV20 | NR | NR | NR | NR | NR | NR | P | F | TET-TMP-SMX-STR | (+) | - | - | (+) | *dfra17, aadA5* |
| COV21 | NR | NR | NR | NR | NR | NR | P | F | TET-SMX | + | - | (+) | (+) | - |
| COV22 | NR | NR | NR | NR | NR | NR | P | F | FQ-TET-TMP-SMX-STR-KAN | + | - | + | + | - |
| COV23 | NR | NR | NR | NR | NR | NR | P | E | FQ-TET-SMX- | + | - | + | + | - |
| COV24 | NR | NR | NR | NR | NR | NR | P | C | FQ-TET-TMP-SMX | *+* | *-* | - | + | *dfra17, aadA5* |
| COV25 | NR | NR | NR | NR | NR | NR | P | E | TET-SMX | + | - | (+) | (+) | - |
| COV26 | NR | NR | NR | NR | NR | NR | P | C | FQ-TET-TMP-SMX | + | - | (+) | (+) | - |
| COV27 | NR | NR | NR | NR | NR | NR | P | F | FQ-TET-TMP-SMX-STR | + |  | + | - | - |
| COV28 | NR | NR | NR | NR | NR | NR | P | F | FQ-TET-SMX | +^i^  -^j^ | -^i^  + ^j^ | +^i^  - ^j^ | +^i^  - ^j^ | - ^i^  - ^j^ |
| COV29 | NR | NR | NR | NR | NR | NR | P | C | FQ-TET-TMP-SMX-STR-KAN | + | - | + | + | - |
| COV30 | NR | NR | NR | NR | NR | NR | P | B2 | TET-SMX | + | - | + | + | - |
| COV31 | NR | NR | NR | NR | NR | NR | P | B2 | FQ-TET-SMX | + | - | + | + | - |
| COV32 | NR | NR | NR | NR | NR | NR | P | F | FQ-TET-SMX | + | - | + | + | - |
| COV33 | NR | NR | NR | NR | NR | NR | P | F | FQ-TET-SMX | + | - | + | + | - |

NR: not reported. NA/ not applicable (no ESCR transformant could be obtained)

^a^Gallus type: C, conventional; E, export; O, organic

^b^*département* of farm and slaughterhouse

^c^age at sampling (in days)

^d^treatment of the flock (AMP: ampicillin, AMX: amoxicillin, COL: colistin, DOXY: doxycycline, FQ: fluoroquinolone, SXT: trimethoprime-sulfonamides)

^e^healthy or from pathology

^f^resistance other than ESC resistance (including ampicillin and cefotaxime for all isolates, cefoxitin for COV1, COV9, COV28 and COV32), TET: tetracycline, SMX: sulfamethoxazole, CIP: ciprofloxacin, TMP: trimethoprim, STR: streptomycin, GEN: gentamicin, KAN: kanamycin

^g^no transformant was obtained for COV8

^h^ARG: antimicrobial resistance gene

^i^ARG borne by pCOV28A

^j^ARG borne by pCOV28B

+: whole gene detected; (+): a part of gene detected; -: gene not detected
