# Supplementary material for: Characterization of plasmids harboring blaCTX-M and blaCMY genes in E. coli from French broilers
Source: PLoS One. 2018 Jan 23;13(1):e0188768. doi: 10.1371/journal.pone.0188768 (PMC5779644; doi:10.1371/journal.pone.0188768)
Supplement: S2 Table — BioProject number (PRJNA387700): the BioSample numbers are provided bellow. (DOCX) [file pone.0188768.s003.docx]

**S2 Table. Accession numbers**

BioProject number (PRJNA387700): the BioSample numbers are provided bellow.

| PacBio | DH5alpha *Escherichia coli* | SAMN07197446 |
| --- | --- | --- |
|  | pCOV24 | SAMN07197432 |
| MiSeq | pCOV1 | SAMN07197409 |
|  | pCOV2 | SAMN07197410 |
|  | pCOV3 | SAMN07197411 |
|  | pCOV4 | SAMN07197412 |
|  | pCOV5 | SAMN07197413 |
|  | pCOV6 | SAMN07197414 |
|  | pCOV7 | SAMN07197415 |
|  | pCOV9 | SAMN07197417 |
|  | pCOV10 | SAMN07197418 |
|  | pCOV11 | SAMN07197419 |
|  | pCOV12 | SAMN07197420 |
|  | pCOV13 | SAMN07197421 |
|  | pCOV14 | SAMN07197422 |
|  | pCOV15 | SAMN07197423 |
|  | pCOV16 | SAMN07197424 |
|  | pCOV17 | SAMN07197425 |
|  | pCOV18 | SAMN07197426 |
|  | pCOV19 | SAMN07197427 |
|  | pCOV20 | SAMN07197428 |
|  | pCOV21 | SAMN07197429 |
|  | pCOV22 | SAMN07197430 |
|  | pCOV23 | SAMN07197431 |
|  | pCOV25 | SAMN07197433 |
|  | pCOV26 | SAMN07197434 |
|  | pCOV27 | SAMN07197435 |
|  | pCOV28A | SAMN07197436 |
|  | pCOV28B | SAMN07197437 |
|  | pCOV29 | SAMN07197438 |
|  | pCOV30 | SAMN07197439 |
|  | pCOV31 | SAMN07197440 |
|  | pCOV32 | SAMN07197441 |
|  | pCOV33 | SAMN07197442 |
