# Supplementary material for: Characterization of plasmids harboring blaCTX-M and blaCMY genes in E. coli from French broilers
Source: PLoS One. 2018 Jan 23;13(1):e0188768. doi: 10.1371/journal.pone.0188768 (PMC5779644; doi:10.1371/journal.pone.0188768)
Supplement: S3 Table — (DOCX) [file pone.0188768.s004.docx]

**S3 Table. Characteristics of sequenced plasmids**

| ESCR gene | Plasmid | Cumulative contig size (bp) | Number of contigs | Replicon type | pMLST | Addiction systems |
| --- | --- | --- | --- | --- | --- | --- |
| *bla*_CTX-M-1_ | pCOV2 | 113,609 | 5 | IncI1 99.30%: G57A | ST3 100% | ParE-RelB |
|  | pCOV3 | 119,904 | 13 | IncI1 99.30%: G57A | ST3 100% | - |
|  | pCOV4 | 111,729 | 1 | IncI1 99.30%: G57A | ST3 100% | ParE-RelB |
|  | pCOV5 | 110,438 | 1 | IncI1 99.30%: G57A | ST3 100% | ParE-RelB |
|  | pCOV6 | 171,865 | 31 | IncI1 99.30%: G57A | ST3 100% | ParE-RelB |
|  | pCOV7 | 112,473 | 3 | IncI1 99.30%: G57A | ST3 100% | ParE-RelB |
|  | pCOV10 | 120,707 | 5 | IncI1 99.30%: G57A | ST3 100% | ParE-RelB |
|  | pCOV11 | 115,593 | 1 | IncI1 99.30%: G57A | ST3 100% | ParE-RelB |
|  | pCOV12 | 110,502 | 1 | IncI1 99.30%: G57A | ST3 100% | ParE-RelB |
|  | pCOV13 | 119,524 | 7 | IncI1 99.30%: G57A | ST3 100% | ParE-RelB |
|  | pCOV14 | 124,476 | 11 | IncI1 99.30%: G57A | ST3 100% | - |
|  | pCOV15 | 108,044 | 1 | IncI1 99.30%: G57A | ST3 100% | ParE-RelB |
|  | pCOV16 | 107,834 | 1 | IncI1 99.30%: G57A | ST3 100% | ParE-RelB |
|  | pCOV17 | 107,875 | 1 | IncI1 99.30%: G57A | ST3 100% | ParE-RelB |
|  | pCOV18 | 107,747 | 1 | IncI1 99.30%: G57A | ST3 100% | CcdB-CcdA / COG5302  ParE-RelB |
|  | pCOV19 | 99,246 | 12 | IncI1 99.30%: G57A | ST3 100% | - |
|  | pCOV20 | 125,378 | 27 | IncI1 99.30%: G57A | ST3 100% | - |
|  | pCOV21 | 117,692 | 18 | IncI1 99.30%: G57A | ST3 100% | - |
|  | pCOV22 | 107,693 | 1 | IncI1 99.30%: G57A | ST3 100% | ParE-RelB |
|  | pCOV23 | 112,743 | 6 | IncI1 99.30%: G57A | ST3 100% | ParE-RelB |
|  | pCOV24 | 131,672 | 1 | IncI1 99.30%: G57A | ST3 100% | ParE-RelB |
|  | pCOV25 | 98,252 | 13 | IncI1 99.30%: G57A | *ardA* not found  *pilL_2, repI1_2, sogS_1* and *trbA_4* | - |
|  | pCOV26 | 100,387 | 12 | IncI1 99.30%: G57A | ST3 100% | - |
|  | pCOV27 | 104,843 | 2 | IncI1 99.30%: G57A | ST3 100% | ParE-RelB |
|  | pCOV28A | 110,520 | 1 | IncI1 99.30%: G57A | ST3 100% | ParE-RelB |
|  | pCOV29 | 114,855 | 3 | IncI1 99.30%: G57A | ST3 100% | ParE-RelB |
|  | pCOV30 | 107,686 | 1 | IncI1 99.30%: G57A | ST3 100% | ParE-RelB |
|  | pCOV31 | 105,870 | 1 | IncI1 99.30%: G57A | ST3 100% | ParE-RelB |
|  | pCOV32 | 110,588 | 1 | IncI1 99.30% : G57A | ST3 100% | ParE-RelB |
|  | pCOV33 | 107,635 | 1 | IncI1 99.30% : G57A | ST3 100% | ParE-RelB |
| *bla*_CMY-2_ | pCOV1 | 91,138 | 6 | B/O/K/Z 97.99%:  G6A, T10C, C135G |  | - |
|  | pCOV9 | 110,249 | 2 | B/O/K/Z 97.32%: G6A, T10C, G13A, C135G |  | RelE-StbD |
|  | pCOV28B | 202,796 | 12 | FIA, 100% 384/388 FIB 99.56%,  FIC/FII 95.59% | F18:A6:B42 | VagC/ SpoVT_AbrB/ MazE-VapC/ PIN |
